# Lnc-SGK1 induced by *Helicobacter pylori* infection and highsalt diet promote Th2 and Th17 differentiation in human gastric cancer by SGK1/Jun B signaling

**DOI:** 10.18632/oncotarget.7823

**Published:** 2016-03-01

**Authors:** Yongliang Yao, Qingbo Jiang, Lixing Jiang, Jianhong Wu, Qinghui Zhang, Jianjun Wang, Huang Feng, Panpan Zang

**Affiliations:** ^1^ Department of Clinical Laboratory, The First People's Hospital of Kunshan, Affiliated to Jiangsu University, Kunshan, Jiangsu, China; ^2^ Department of Clinical Laboratory, The Third Affiliated Hospital of Suzhou University, Changzhou, Jiangsu, China; ^3^ Department of Clinical Laboratory, Wujin Hospital Affiliated to Jiangsu University, Changzhou, Jiangsu, China

**Keywords:** gastric cancer, high-salt diet, Helicobacter pylori infection, serum and glucocorticoid-inducible kinase, LncRNA

## Abstract

Serum and glucocorticoid-inducible kinase (SGK) 1can be triggered in several malignancies. Most research on SGK1has focused on its role in cancer cells, and we sought to investigate its potential upstream non-coding RNA nominated as Lnc-SGK1, and their expression and diagnostic value in T cells in human gastric cancer (GC). Excessive expression of Lnc-SGK1 and SGK1 were observed in T cell either within the tumor or peripheral T cells, and furthermore associated with *Helicobacter pylori* infection and high-salt diet (HSD). Within T cells, *Helicobacter pylori* (*Hp*) infection and high-salt dietcan up-regulated SGK1 expression and in turn enhance expression of Lnc-SGK1 through JunB activation. And expression of Lnc-SGK1 can further enhance transcription of SGK1 through *cis* regulatory mode. Lnc-SGK1 can induce Th2 and Th17 and reduce Th1 differentiation via SGK1/JunB signaling. Serum Lnc-SGK1 expression in combination with *H. pylori* infection and/or HSD in T cells was associated with poor prognosis of GC patients, and could be an ideal diagnostic index in human GC.

## INTRODUCTION

Serum and glucocorticoid-inducible kinase (SGK)1is an AGC protein kinase of the SGK family, which can be triggered in response to a variety of physiological and pathological stimuli [1]. Physiologically, SGK1 can be triggered by hyperosmotic cell shrinkage such as dehydration [2] and a modest increase of extracellular salt concentration [3]; and several hormones and mediators (cytokines), such as glucocorticoids [4], mineralocorticoids [5] transforming growth factor β [6] and interleukin (IL)-6 [7]. SGK1 transcription can be regulated by different signaling including cytosolic Ca^2+^, cyclic AMP, stress-activated protein kinase (SAPK2, p38 kinase), protein kinase C, and extracellular signal-regulated kinase (ERK)1/2, and be activated by insulin, insulin-like growth factor (IGF)1, and hepatic growth factor (HGF) [8].

SGK1 can also be enhanced in some pathological conditions, including diabetes, dialysis, glomerulonephritis, liver cirrhosis and malignancies [1]. High levels of SGK1 expression have been observed in several tumors [8, 9], including colon cancer [10], myeloma [7], ovarian tumor [11], and non-small cell lung cancer [12]. SGK1 may support survival of tumor cells by counteracting apoptosis via phosphorylation, and thus inhibits glycogen synthase kinase 3, which downregulate oncogenic β-catenin [13, 14].

However, the above description concerning the role of SGK1 was discovered within tumor cells, and the role of SGK1 in the tumor microenvironment is less reported. A few studies have addressed the role of SGK1 in T cell differentiation. SGK1 upregulates pathogenic IL-23-dependent IL-17-producing CD4^+^ helper T cells (Th17 cells), which play a decisive role in autoimmune disease and malignancy [15, 16]. Recently, Heipamp et al. have found that after activation by mammalian target of rapamycin complex 2, SGK1 promotes Th2differentiation by negatively regulating degradation of the transcription factor JunB mediated by the E3 ligase Nedd4-2. Simultaneously, SGK1 repressed sproduction of interferon (IFN)-γ by controlling expression of the long isoform of the transcription factor TCF-1 [17]. Although biological feature of SGK1 have been addressed systemically, the up-stream regulation of SGK1 was rare documented, especially those non-coding RNA.

The long non-coding RNAs (lncRNAs) were reported as a biomarker for predicting survival, metastasis, and in the diagnosis of multiple diseases [18, 19]. The functional effects of lncRNA have been widely recognized, including regulating gene expression through modulation of chromatin remodeling, controlling of gene transcription, post-transcriptional mRNA processing, protein function or localization, and intercellular signaling [20–22]. To characterize the relationships of lncRNA and protein coding RNA, researchers have defined a “Flank10kb” theory which means lncRNA genes mapping within 10 kb of a known annotatedgene (NCBI RefSeq or UCSC Known Genes) on thesame genomic strand. The overlapped lncRNA tended to interact with protein through *acis*-regulatory process. Interestingly, we found a lncRNA (LOC105378009) located in the upstream of SGK1 promoter region within 10kb space, therefore, in the present study, we sought to investigate the role of SGK1 within T cells of patients with gastric cancer (GC), and the association between SGK1 and LOC105378009 as well as the characteristics of GC and their potential prediction value for diagnosis or prognosis.

## RESULTS

### LOC105378009 promoted the SGK1 expression in a *cis*-regulation

**Figure 1:**
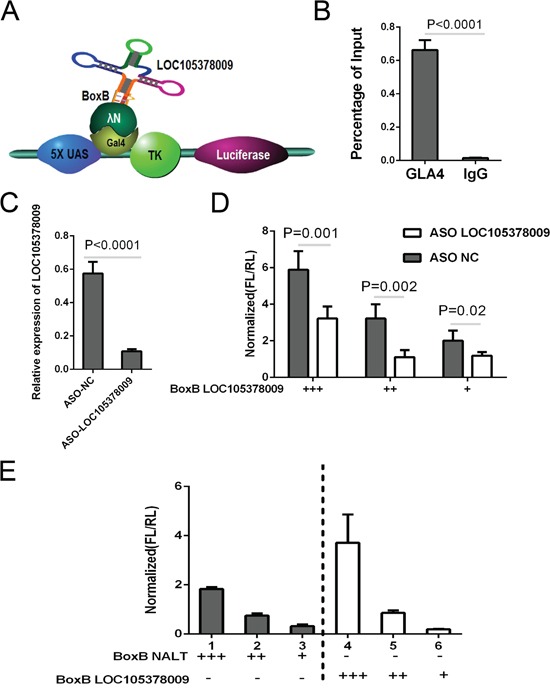
LOC105378009 promoted the SGK1 expression in a *cis*-regulation **A.** The schematic system for Gal4-λN/BoxB reporter system. **B.** Percentage of the binding ability of Gal4-λN fusion by ChIP. **C.** The knock-down of LOC105378009 by ASO technology. **D.** Reporter assay showing relative reporter gene activity when BoxB-NALT was co-transfected with either control or ASO targeting LOC105378009. **E.** The luciferase reporter activity in experiments where BoxB-tagged LOC105378009(right) or Lnc-NALT (left) were co-transfected with Gal4-lN. Data was normalized to Ranilla luciferase. Data presented as the Mean ± SEM.

Since the upregulation of SGK1 has been identified causing the Th2/Th17 shift, we further aimed to investigate the potential simulating factors of SGK1. Bioinformatics analyses recently have reported an underlying method to discover the putative candidate genes in which a “Flank10kb” analysis was described [23]. The novel analysis revealed that over 65% of lncRNA genes were located within 10 kb of known, primarily protein-coding genes. They suggested that *cis*-regulatory relationships may exist between lncRNAs and known genes. According to the bioinformatics analyses, LOC105378009 was located upstream of SGK1, with a spacing of about 5 kb (Supplementary Figure 1) which was within the Flank10K class. We proposed that the long non-coding RNA, LOC105378009, located in the up-stream of SGK1, might regulate the expression SGK1 through a *cis*-regulation. Gal4-λN/BoxB reporter system was employed as described previously to explore whether LOC105378009 regulated SGK1 as a *cis*-pattern [24]. In this system, the BoxB RNA stem loop is fused to LOC105378009; LUNAR1 was used as a positive control. The plasmid encoding a TK-luciferase gene under the control of five GAL4 UAS sites was co-transfected with plasmids encoding GAL4-λNpeptide as described above (Figure 1A). Ranilla luciferase was regarded as control in this system. The binding of Gal4-λN fusion was confirmed firstly (Figure 1B). Luciferase activity after co-transfection of the system indicated that tethering LOC105378009 to this reporter gene could stimulate transcription of the reporter to a similar degree as LUNAR1 indicating that LOC105378009 could function as a transcription activator (Figure 1C).

Further antisense oligonucleotides (ASO) targeting LOC105378009 was designed, we found that the activation affection was significantly reduced comparing to control group (Figure 1D, 1E). From this study we confirmed that there is a Lnc-RNA, LOC105378009 located in the up-stream of SGK1, which can promote SGK1 expression via cis-regulatory mode, thus we nominate this lnc-RNA as Lnc-SGK1.

### Lnc-SGK1 and SGK1 expression was enhanced in HSD and *Helicobacter pylori* infection GC patients

**Figure 2:**
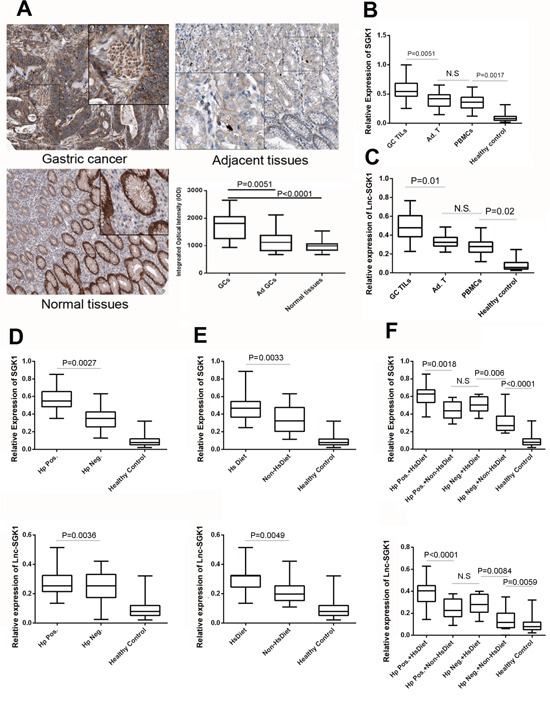
Lnc-SGK1 and SGK1 expression was enhanced in human GC, especially in infiltrating T cells **A.** Immunohistochemicalstaining of SGK1 in human GC, adjacent tissues and normal gastric cancers. Average integrated optical density was obtained by analyzing five fields for each slide evaluated by Image-Pro Plus software (version 5.0) for immunohistochemistry of SGK1 (*n* = 245 for Gastric Cancer tissues (GCs) and Adjacent tissues (Ad GCs), *n* = 118 for Normal tissues). **B, C.** Expression of SGK1 and Lnc-SGK1 was determined by real-time PCR in infiltrating T cells within the tumor (GC TILs), infiltrating T cells within adjacent normal tissues (Ad TILs), PBMCs of GC patients (PBMCs), and PBMCs from healthy controls (Healthy control). Relative expression of SGK1 and lnc-SGK1 in TILs from GC patients with or without *H. pylori* infection **D.**, HSD **E.**, and combined factors **F**. The upper panel indicated the SGK1 expession while the lower indicated lnc-SGK1. All data presented as mean±SD, **P < 0.01 by unpaired Student's *t* test.

To investigate the distribution of Lnc-SGK1 and SGK1 in human GC, 245 tumor tissues and adjacent normal tissues were analyzed. GC and normal tissues both expressed SGK1, especially in the parenchymal cells. However, expression of SGK1 was increased significantly in GC compared with both tumor adjacent tissues and normal tissues (Figure 2A). It was attractive that the difference in SGK1 expression in non-parenchymal cells between GC and normal tissues was evident (Figure 2A).

Next, Expression of both SGK1 and Lnc-SGK1 was determined by real-time PCR. Lnc-SGK1 and SGK1 transcription was significantly higher in GC tumor infiltration cells (TILS) than adjacent normal tissue and peripheral blood mononuclear cells (PBMCs). A significant difference was also found for Lnc-SGK1 and SGK1 expression between the healthy control and GC PBMCs groups (Figure 2B, 2C).

SGK1 and Lnc-SGK1 expression in peripheral T cell of patients with *H. pylori* infection (Hp Pos., 187 cases) was higher than in those without infection (Hp. Neg., 58 cases) (Figure 2D). Similarly, peripheral T cells from GC patients with HSD (HsDiet 118 cases) also have significantly stronger SGK1 and Lnc-SGK1 expression than patients without HSD (Non-HsDiet 127 cases) (Figure 2E). In general, *H. pylori* infection and HSD are risk factors for human GC related to SGK1 expression according to the clinical data.

Furthermore, the 245 GC patients were subdivided into four groups according to the two risk factors: *H. pylori* infection and HSD: Hp.Pos+HsDiet: 85 cases; Hp.Pos+Non-HsDiet: 102cases; Hp.Neg+HsDiet: 33 cases; and Hp.Neg+Non-HsDiet: 25 cases. Expression of SGK1 in T cells in each group was compared. Hp.Pos+HsDiet patients had the highest Lnc-SGK1 and SGK1 expression among these four groups. There was no significant difference between Hp.Pos.+Non-HsDiet and Hp.Neg.+HsDiet, which might implied that *H. pylori* infection and HSD contribute equally to overexpression of SGK1 and Lnc-SGK1. The Hp.Neg.+Non-HsDiet group had the lowest expression of lnc-SGK1and SGK1, while still significantly higher than in the healthy controls (Figure 2F).

In summary, T cell SGK1 expression in human GC is related to *H. pylori* infection and HSD. However, what high SGK1 expression in T cells implies requires further investigation.

### High salt and *H. pylori* lysate can up-regulate SGK1 and Lnc-SGk1 expression

**Figure 3:**
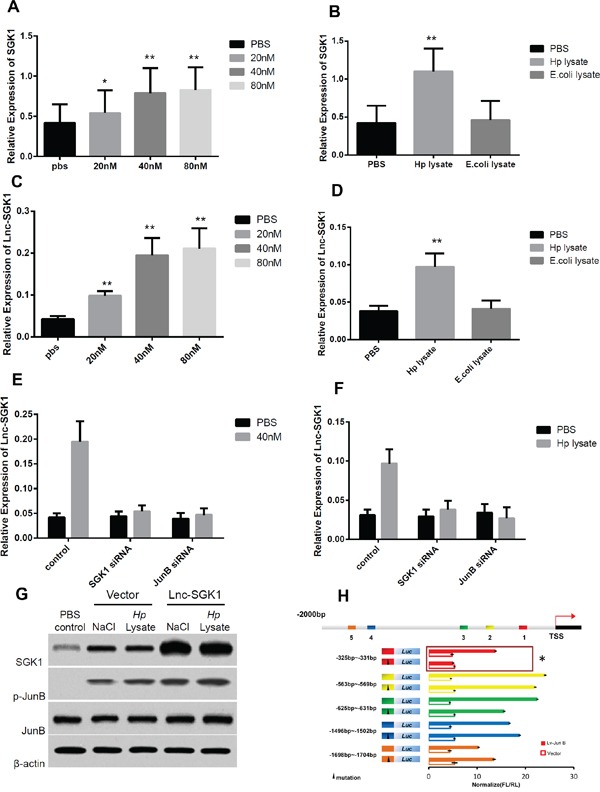
High salt and Hp lysate can up regulate SGK1 and Lnc-SGk1 expression **A-D.** T cells were treated with either different concentration of NaCl or Hp and E. coli lysate and then the expression of both SGK1 and Lnc-SGK1 was detected by real-time PCR. **E-F.** T cells were modified with specific siRNA to SGK1 and JunB, and next the T cells were treated with 40nM NaCl and Hp lysate. **G.** T cells was transected with Lnc-SGK1 and treated with 40nM NaCl and Hp lysate the expression of SGK1 and phosphorated JunB was detected by western-blot. **H.** Luciferase reporter analysis was performed using promoter 2Kb ahead of start code; mutagenesis was performed based on different JunB binding sites. Each experiment was performed three times separately, P < 0.05 was considered as significance.

In order to investigate high salt diet and *H. pylori* infection whether have effect on SGK1 as well as Lnc-SGK1 expression in T cells, *in vitro* experiment were designed. First, SGK1 transcription increased significantly in T cells treated with different concentration of NaCl (Figure 3A), in 20nM the SGK1 expression raised slightly, when the concentration up to 40nM, more dramatically increase of SGK1 was observed however, no apparent change was found when the concentration was up to 80nM. *H. pylori* infection can similarly increase SGK1 expression (Figure 3B), when T cell was co-cultured with *H. pylori* lysate, transcription of SGK1 increased significantly, and moreover, the stimulation was *H. pylori* specific, because lysate of *E.coli* cannot up-regulate SGK1 transcription.

Meanwhile, the effect of NaCl and *H. pylori* infection on transcription of Lnc-SGK1 were also been detected, similar to SGK1, increased NaCl concentration and *H. pylori* lysate can specifically increase the transcription of Lnc-SGK1 (Figure 3C, 3D). In order to investigate whether up-regulation of Lnc-SGK1 was SGK1/JunB dependent, specific siRNA was designed. Lnc-SGK1 expression was accessed with the condition of high salt and *Hp* infection. When SGK1 or JunB was knockdown by their specific siRNA, enhanced expression of Lnc-SGK1 was not observed with the treatment of either high salt or *Hp* infection (Figure 3E, 3F).

Moreover, as we presented above, existence of Lnc-SGK1 can up-regulate SGK1 transcription by a *cis* regulatory mode, which was further confirmed by western-blot, plus SGK1 can activate JunB which is a transcriptional factor for Lnc-SGK1 (Figure 3G, 3H and supplementary Figure 2). Thus, we can find a loop here, stimulus from outside such as high salt and *H. pylori* infection can enhance expression of SGK1 and activate JunB, activation of JunB can in turn up-regulate transcription of Lnc-SGK1, and enhancement of Lnc-SGK1 can not only increase SGK1 expression but also enrich the activation of JnuB, especially with the treatment of NaCl and *H. pylori* infection (Figure 3E, 3F). The effect of Lnc-SGK1 in T cell should be further studied.

### Lnc-SGK1 can promote Th2 and Th17 differentiation through SGK1/JunB signaling

**Figure 4:**
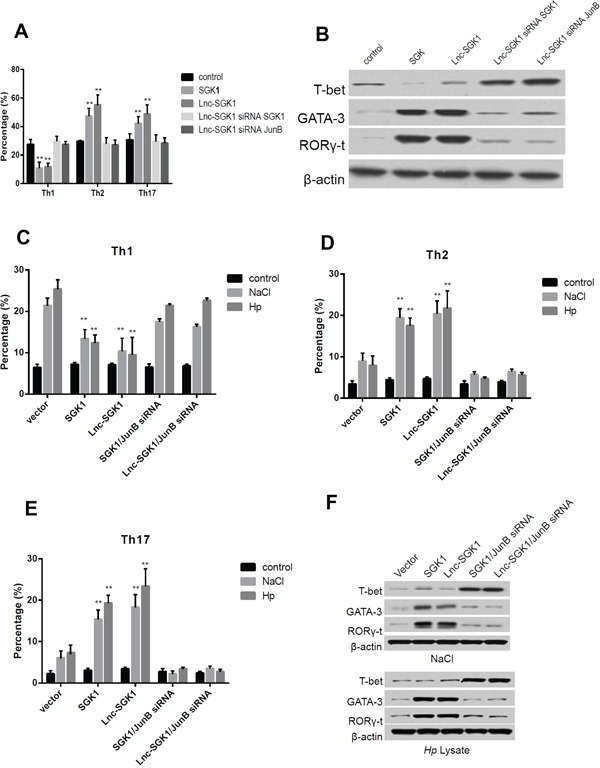
Lnc-SGK1 can promote Th2 and Th17 differentiation through SGK1/JunB signaling **A.** Polarization stimulations were carried out to Th1 Th2 and Th17 respectively, T cells was transected indicated in the figure, the percentage of each cells was determined by flow cytometry. **B.** Transcriptional factor of different type of T help cell were determined by western-blot in polarization stimulated T cells modified indicated in the figure. T cells modified indicating in the figure and co-culture with either NaCl and Hp lysate, the percentage of Th1, Th2 and Th17 were respectively determined by flow cytometry and presented in figure **C**, **D** and **E.** Moreover, the transcriptional factors of T helper cell were detected by western-blot and further presented in **F.** Each experiment was performed three times separately, P < 0.05 was considered as significance.

In order to investigate the effect of Lnc-SGK1 on T help cell differentiation, polarization stimulation was performed according to previous condition. First it was similar to previous publication that overexpression of SGK1 in T cells can promote Th2 and Th17 differentiation meanwhile the differentiation of Th1 was significantly suppressed (Figure 4A). Next, overexpression of Lnc-SGK1 further enhanced Th2 and Th17 differentiation by up-regulation of SGK1. In order to investigate that the effect of SGK1 was depend on SGK1/JunB signaling, SGK1 and JunB was respectively knocked down by siRNA, interestingly, percentage of Th1 cells increased significantly and have no difference compared to control group, while the percentage of both Th2 and Th17 went down significantly (Figure 4A). Furthermore, the hall mark transcription factors were detected by western-blot which can verified the results of polarization stimulation. T-bet increased with either SGK1 or JunB were knocked down and GATA3 and RORγt were decreased even when Lnc-SGK1 was overexpressed (Figure 4B). All above might indicated that Lnc-SGK1 could promote Th2 and Th17 cell differentiation just depended on SGK1/JunB signaling.

In order to further investigate the roles of Lnc-SGK1 on T cell differentiation during high salt concentration or *H. pylori* infection, the T cells was treated with 40nM Nacl and *H. pylori* lysate. The percentage of Th2 and Th17 increased and Th1 decreased, in both T cells transfected with Lnc-SGK1 and SGK1, however, when JunB was knocked down by siRNA, the increase of Th2 and Th17 was attenuated and the percentage of Th1 increased (Figure 4C–4E), which might implied that enhancement of both Lnc-SGK1 and SGK1 on Th2 and Th17 differentiation was depended on JunB activation. And last, the transcriptional factor of each Th cells was determined by western-blot, and indicated the same result as flow cytometer (Figure 4F).

### Serum Lnc-SGK1 were negative prognostic indicators and ideal diagnostic indicators in GC

**Figure 5:**
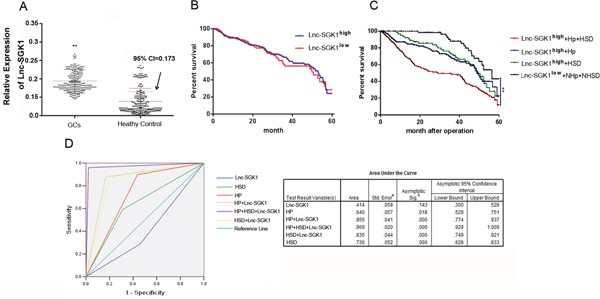
*H. pylori* infection and HSD induced SGK1 expression in T cells was an ideal diagnostic and prognostic indicator of GC **A.** Expression of SGK1 in PBMCs of GC patients and healthy controls. The threshold discriminating normal from elevated systemic levels was obtained using 95% CI in the healthy controls. **B.** The Kaplan–Meier curve for the overall survival of patientswas presented by using different indicators, such as SGK1 expression. **C.** SGK1 expression combined with *H. pylori* infection and HSD. **D.** ROC curve analysis was conducted for discrimination between cases and controls by the threefactors independently or in combination. AUC is indicated under the curve.

Because serum lncRNA was reported to be indicator of various diseases recently, we also accessed lnc-SGK1 in the serum of GC patients. Assessment of the 95% confidence interval (CI) in the healthy control group indicated that 0.173 was the threshold from discriminating normal from elevated systemic levels in the serum. The 245 GC patients were divided into two groups accordingly: ≤0.173 (93cases, SGK1^Low^) and >0.173 (152 cases, SGK1^High^). The overall survival rate was investigated based on follow-up data (Figure 5A). There was no significant difference in overall survival (*P* = 0.704) between these two groups. Therefore, Lnc-SGK1 expression in serum of GC patients is not an ideal prognostic indicator in GC (Figure 5B).

The patients were divided into groups according to Lnc-SGK1 expression in PBMCs, *H. pylori* infection and HSD. Patients with high/low Lnc-SGK1 expression combined with *H. pylori* infection and/or HSD were divided into six groups: Lnc-SGK1^high^+Hp+HSD (*n* = 59), Lnc-SGK1^high^+Hp (*n* = 48), Lnc-SGK1^high^+HSD (*n* = 45), Lnc-SGK1^low^+NHp+NHSD (*n* = 65), Lnc-SGK1^low^+NHp (*n* = 12) and Lnc-SGK1^low^+NHSD (*n* = 16). We assessed the 5-year survival rate in the four groups with >40 cases. The 5-year survival rate in the Lnc-SGK1^high^+Hp+HSD group was only 10.27%, which was significantly lower than in the other three groups. There was no significant difference in the 5-year survival rates between the Lnc-SGK1^high^+Hp and Lnc-SGK1^high^+HSD groups. The 5-year survival rate in the Lnc-SGK1^low^+NHp+NHSDgroup was significantly higher than in the other three groups (Figure 5C).

ROC curve analysis was performed to assess the diagnostic sensitivity and specificity of SGK1 expression in GC by using risk score functions. The diagnostic value of Lnc-SGK1 expression in PBMCs of GC patients was not ideal, with an area under the curve (AUC) of 0.414(95% CI: 0.300–0.528). The diagnostic value was enhanced by combining with either *H. pylori* infection or HSD. The sensitivity and specificity was the highest using Lnc-SGK1 expression in PBMCs combined with *H. pylori* infection and HSD (AUC: 0.969 95% CI:0.929–1.009), and decreased using Lnc-SGK1+Hp and Lnc-SGK1+HSD (Lnc-SGK1+Hp, AUC: 0.855, 95% CI:0.774–0.937; Lnc-SGK1+HSD, AUC: 0.835, 95% CI:0.749–0.921). However, sensitivity and specificity were better than for *H. pylori* infection and HSD, which are well-known diagnostic indices of GC (*H. pylori*, AUC: 0.730, 95% CI:0.628–0.833; HSD, AUC: 0.640, 95% CI:0.529–0.751) (Figure 5D). These results indicated that *H. pylori* infection and HSD induced SGK1 expression were ideal indicators in screening of GC.

## DISCUSSION

GC is defined as cancer that forms in the tissues lining the stomach. Globally, GC is the fifth leading cause of cancer and the third leading cause of cancer mortality, comprising 7% of cases and 9% of deaths. In 2012 GC occurred in 950,000 people and caused 723,000 deaths [25]. The most common cause is infection by *H.pylori*, which accounts for >60% of cases [26, 27]. HSD is also related to GC and *H. pylori* infection. Salt has been found to increase the growth and action of *H. pylori*, thus increasing the risk of cancer [28, 29]. However, in the present study, we found another link between HSD and tumorigenesis of GC. Both *H. pylori* infection and HSD were related to Lnc-SGK1 and SGK1 expression in T cells of GC.

*H. pylori* is specifically adapted to survive in the gastric environment, resulting in the development of gastritis, and recruitment of neutrophils, followed by B and T lymphocytes, macrophages and plasma cells. Consequently, large amounts of reactive oxygen or nitrogen species, involved in epithelial cell damage and carcinogenesis, are generated. However, inflammation is variable, including Th1, Th2, Th17 and regulatory T cell responses [30–32]. Although *H. pylori*-induced chronic atrophic gastritis is characterized by marked infiltration of Th1 cells, However, Th2 and Th17 were more seriously associated with development of GC. Besides the Th1 response, the Th2 response was still observed in human GC, and was related to the development of *H. pylori*-related cancer. Kido et al. have reported that *H. pylori* can directly trigger epithelial cells to produce thymic stromal lymphopoietin, and in turn mediate dendritic cell activation, which is involved in the Th2 responses that trigger B-cell activation in *H. pylori*-induced gastritis. In our study, the Th2 and Th17 response was confirmed to be dominant in *H. pylori*-positive gastric cancer, which is linked to Lnc-SGK1 and SGK1 was overexpression. In addition, Although T cell Lnc-SGK1 expression was related to tumor size and metastasis, Lnc-SGK1 alone was not sufficient to determine the prognosis of GC. However, Lnc-SGK1 expression in T cells could be a good prognostic indicator in GC when combined with other factors: either *H. pylori* infection or HSD.

HSD can increase the carcinogenesis induced by enhanced gastritis and CagA expression. HSD causes severe gastritis, high gastric pH, increased parietal cell loss, increased gastric expression of IL-1β, and decreased gastric expression of hepcidin and hydrogen potassium ATPase (H,K-ATPase), compared to individuals on a regular diet [33]. These results indicated that HSD potentiates the carcinogenic effects of cagA^+^ *H. pylori* strains [34]. In the present study, we found that Lnc-SGK1, *H. pylori* infection and HSD can serve as an ideal diagnostic indicator in GC. Expression of Lnc-SGK1 was associated with Th2 and Th17 response in GC patients with *H. pylori* infection and/or HSD. Previous studies have suggested the mechanism of the effect of SGK1 on T cell differentiation. Heikamp et al. (2004)have shown that expression of SGK1 in T cells promotes Th2 differentiation. SGK1 is indispensible in upregulating pathogenic IL-23-dependent Th17 cells [16].

In summary, we investigated the expression of Lnc-SGK1 in human GC, and showed that Lnc-SGK1 in combination with *H. pylori* infection and HSD can serve as ideal diagnostic indicator in GC.

## MATERIALS AND METHODS

### Patients

A total of 245 paired GC and adjacent tissues were obtained at the time of surgical resection between January 2009 and December 2015 at the People's Hospital of Kunshan City, China. Samples were collected in accordance with the principles expressed in the Declaration of Helsinki. Written documented informed consent for gene expression analyses of all tissues was obtained from all patients prior to surgery or endoscopy examination. The study and consent procedures were approved by the Ethics Committee of the First People's Hospital of Kunshan City. Judgment of high-salt diet (HSD) intake of GC patients was obtained by questionnaire. In brief, GC patients who whether belong to HSD population was based on Nutrition and Health Survey of Chinese in 2002 published by Chinese ministry of Healthy, people who intake salt over 10g was classified into HSD patients. The patients whether infected with Hp can be determined by Carbon 13 breath test. The detailed clinical characteristics of the 245 GC patients are listed in Table 1.

**Table 1:** Clinical characteristics of 245 GC patients

Patient demographics	Count
**Age, y (range)**	53(31–85)
**Sex**	
Male	127
Female	118
**Main location**	
U	131
ML	114
**Size, cm (range)**	
>5	137
<5	108
**Depth of invasion**	
T1/T2	126
T3/T4	119
**Lauren classification**	
Intestinal	119
Diffuse	126
**Lymph node metastasis**	
Positive	147
Negative	98
***H.pylori* infection**	
Positive	187
Negative	58
**High salt diet intake**	
Positive	118
Negative	127
**Stage**	
I	79
II	45
III	43
IV	78

### Isolation and culture of leukocytes and peripheral blood mononuclear cells (PBMCs) from GC patients

Fresh GC tissues were washed twice in RPMI 1640. Fatty, connective and necrotic tissue was removed. Tissues were minced into 1–2-mm pieces in RPMI 1640, transferred into 15- or 50-ml conical tubes, and incubated with triple enzyme digestion medium containing DNase (30 U/ml), hyaluronidase (0.1 mg/ml), and collagenase (1 mg/ml) for 2 hours at room temperature with gentle shaking. Tissues were resuspended in 10 ml RPMI 1640 and filtered through a 70-μm cell strainer (BD Pharmingen). Tissues trapped by the strainer wereplaced into individual wells containing 1 ml T-cell growth medium in a 24-well plate for isolation by T cell isolation kit (Miltenyi Biotec).

PBMCs from GC patients and normal controls were isolated by using Ficoll Paque Plus (GE), according to the manufacturer's instructions, and further isolated by T cell isolation kit (Miltenyi Biotec).

### Quantitative real-time polymerase chain reaction (PCR)

Reverse transcription reactions were performed using the SuperScript First-Strand Synthesis System (Invitrogen), and the RNA templates were treated with DNase to avoid genomic DNA contamination. To determine the relative level of cDNA in the reverse transcribed samples, real-time PCR analyses were performed using an Applied Biosystems 7300 Detection System. The primer sequences used for detecting SGK1 were (5′-3′) forward primer:AGGATGGGTCTGAACGACTTT and reverse primerGCCCTTTCCGATCACTTTCAAG for human SGK1, and the amplification length was 208 bp. Primer for Lnc-SGK1: Forward primer AGAGGACGCAGGAGATTGGA; Reverse primer CAAGGCTGAAGCATCTCCGTA. The amplification length was 198 bp. The primer sequences used for detecting GAPDH were (5′-3′) forward primer:TGTGGGCATCAATGGATTTGG and reverse primerACACCATGTATTCCGGGTCAAT for human GAPDH, and the amplification length was 116 bp, which served as an internal control. The primers were synthesized by Genscript. Real-time PCR reactions were performed in accordance with the instructions of the SYBR Premix Ex Taq kit (Takara). Data were normalized tothe GAPDH levels in the samples.

### Immunohistochemistry

All the tissues were removed and fixed in 4% paraformaldehyde overnight at 4°C, processed, and sectioned at 5-μm thickness. The sectioned slides were stained immunohistochemically for SGK1 (purchased from Abcam) following routine protocols.

### Flow cytometry determination

For intracellular cytokine staining, cells were stimulated at 37° C for 5 hours with a Leukocyte Activation Cocktail (BD Pharmingen). Cells were then stained with surface markers, fixed, and permeabilized with IntraPre Reagent (Beckman Coulter), and finally stained with intracellular markers. Data were acquired on FACSVantage SE and analyzed with CellQuest software. Fluorochrome-conjugated monoclonal antibodies against SGK1, CD3, CD11b, INF-γ, IL-4 and IL-17A were purchased from BD Pharmingen.

### Gal4-λN/BoxB reporter assay

In this system, the BoxB RNA stem loop is fused to LOC105378009, LUNAR1 was used as a positive control [24]. The plasmid encoding a TK-luciferase gene under the control of five GAL4 UAS sites was co-transfected with plasmids encoding GAL4-λN peptide fused to a C-terminal GFP tag, BoxB as described above. Ranilla luciferase was regarded as control in this system. The binding of Gal4-λN fusion was confirmed firstly.

### ASO technology

Antisense oligonucleotides (ASOs) were designed using the IDT Antisense Design Tool (http://www.idtdna.com) using the chimeric 25-mer setting. The top 3 ASOs generated by the design tool were ordered and tested for knockdown efficiency for further investigation. Sequence for the control group in ASO assay was taken from Thomas Trimarchi, et al [24]. For ASO knockdown in BoxB tethering experiments, ASOs were co-transfected with plasmid DNA at 50 nM.

### Dual-luciferase reporter assay

T cell line Jurkat were culture in medium with IL-2 supplied. The Lnc-SGK1 promoter (either wild type or mutant type) luciferase reporter vector (pGL4 packaged) and mock pGL4 vector was added to 3×10^6^ Jurkat cells. After a 24h culture in serum-free conditions and stimuli, luciferase activity was measured by the dual luciferase assay system (Promega, WI, USA), according to the manufacturer's instructions. Data were normalized by the activity of Renilla luciferase.

### Statistical analysis

The results are expressed as mean±SD. Comparisons between two groups were performed using Student's *t*test or Mann–Whitney *U* test, as appropriate. Risk score analysis was performed to investigate the effectiveness of the three-factors signature for tumor occurrence. Frequency tables and receiver operating characteristic (ROC) curves were then used to evaluate the diagnostic effects of the factors. All statistical analyses were performed using SPSS version 13.0, and two-tailed *t* tests were applied to all data unless otherwise specified, with *P* < 0.05 considered statistically significant.

The authors declare no competing financial interests.

## SUPPLEMENTARY FIGURES



## References

[1] Lang F, Voelkl J (2013). Therapeutic potential of serum and glucocorticoid inducible kinase inhibition. Expert opinion on investigational drugs.

[2] Chen S, Grigsby CL, Law CS, Ni X, Nekrep N, Olsen K, Humphreys MH, Gardner DG (2009). Tonicity-dependent induction of Sgk1 expression has a potential role in dehydration-induced natriuresis in rodents. The Journal of clinical investigation.

[3] Nakano M, Hirooka Y, Matsukawa R, Ito K, Sunagawa K (2013). Mineralocorticoid receptors/epithelial Na(+) channels in the choroid plexus are involved in hypertensive mechanisms in stroke-prone spontaneously hypertensive rats. Hypertension research.

[4] Singh PK, Singh S, Ganesh S (2013). Activation of serum/glucocorticoid-induced kinase 1 (SGK1) underlies increased glycogen levels, mTOR activation, and autophagy defects in Lafora disease. Molecular biology of the cell.

[5] Liu G, Liu G, Alzoubi K, Umbach AT, Pelzl L, Borst O, Gawaz M, Lang F (2013). Upregulation of store operated Ca channel Orai1, stimulation of Ca(2+) entry and triggering of cell membrane scrambling in platelets by mineralocorticoid DOCA. Kidney & blood pressure research.

[6] Gao S, Alarcon C, Sapkota G, Rahman S, Chen PY, Goerner N, Macias MJ, Erdjument-Bromage H, Tempst P, Massague J (2009). Ubiquitin ligase Nedd4L targets activated Smad2/3 to limit TGF-beta signaling. Molecular cell.

[7] Fagerli UM, Ullrich K, Stuhmer T, Holien T, Kochert K, Holt RU, Bruland O, Chatterjee M, Nogai H, Lenz G, Shaughnessy JD, Mathas S, Sundan A, Bargou RC, Dorken B, Borset M (2011). Serum/glucocorticoid-regulated kinase 1 (SGK1) is a prominent target gene of the transcriptional response to cytokines in multiple myeloma and supports the growth of myeloma cells. Oncogene.

[8] Lang F, Shumilina E (2013). Regulation of ion channels by the serum- and glucocorticoid-inducible kinase SGK1. FASEB.

[9] Lang F, Stournaras C (2013). Serum and glucocorticoid inducible kinase, metabolic syndrome, inflammation, and tumor growth. Hormones.

[10] Lang F, Perrotti N, Stournaras C (2010). Colorectal carcinoma cells–regulation of survival and growth by SGK1. The international journal of biochemistry & cell biology.

[11] Melhem A, Yamada SD, Fleming GF, Delgado B, Brickley DR, Wu W, Kocherginsky M, Conzen SD (2009). Administration of glucocorticoids to ovarian cancer patients is associated with expression of the anti-apoptotic genes SGK1 and MKP1/DUSP1 in ovarian tissues. Clinical cancer research.

[12] Abbruzzese C, Mattarocci S, Pizzuti L, Mileo AM, Visca P, Antoniani B, Alessandrini G, Facciolo F, Amato R, D'Antona L, Rinaldi M, Felsani A, Perrotti N, Paggi MG (2012). Determination of SGK1 mRNA in non-small cell lung cancer samples underlines high expression in squamous cell carcinomas. Journal of experimental & clinical cancer research: CR.

[13] Su Y, Qadri SM, Cayabyab FS, Wu L, Liu L (2014). Regulation of methylglyoxal-elicited leukocyte recruitment by endothelial SGK1/GSK3 signaling. Biochimica et biophysica acta.

[14] Hagiwara A, Cornu M, Cybulski N, Polak P, Betz C, Trapani F, Terracciano L, Heim MH, Ruegg MA, Hall MN (2012). Hepatic mTORC2 activates glycolysis and lipogenesis through Akt, glucokinase, and SREBP1c. Cell metabolism.

[15] Kleinewietfeld M, Manzel A, Titze J, Kvakan H, Yosef N, Linker RA, Muller DN, Hafler DA (2013). Sodium chloride drives autoimmune disease by the induction of pathogenic TH17 cells. Nature.

[16] Wu C, Yosef N, Thalhamer T, Zhu C, Xiao S, Kishi Y, Regev A, Kuchroo VK (2013). Induction of pathogenic TH17 cells by inducible salt-sensing kinase SGK1. Nature.

[17] Heikamp EB, Patel CH, Collins S, Waickman A, Oh MH, Sun IH, Illei P, Sharma A, Naray-Fejes-Toth A, Fejes-Toth G, Misra-Sen J, Horton MR, Powell JD (2014). The AGC kinase SGK1 regulates TH1 and TH2 differentiation downstream of the mTORC2 complex. Nature immunology.

[18] Cesana M, Cacchiarelli D, Legnini I, Santini T, Sthandier O, Chinappi M, Tramontano A, Bozzoni I (2011). A long noncoding RNA controls muscle differentiation by functioning as a competing endogenous RNA. Cell.

[19] Batista PJ, Chang HY (2013). Long noncoding RNAs: cellular address codes in development and disease. Cell.

[20] Quagliata L, Matter MS, Piscuoglio S, Arabi L, Ruiz C, Procino A, Kovac M, Moretti F, Makowska Z, Boldanova T, Andersen JB, Hammerle M, Tornillo L, Heim MH, Diederichs S, Cillo C (2014). Long noncoding RNA HOTTIP/HOXA13 expression is associated with disease progression and predicts outcome in hepatocellular carcinoma patients. Hepatology.

[21] Hammerle M, Gutschner T, Uckelmann H, Ozgur S, Fiskin E, Gross M, Skawran B, Geffers R, Longerich T, Breuhahn K, Schirmacher P, Stoecklin G, Diederichs S (2013). Posttranscriptional destabilization of the liver-specific long noncoding RNA HULC by the IGF2 mRNA-binding protein 1 (IGF2BP1). Hepatology.

[22] Takahashi K, Yan I, Haga H, Patel T (2014). Long non-coding RNA in liver diseases. Hepatology.

[23] Jia H, Osak M, Bogu GK, Stanton LW, Johnson R, Lipovich L (2010). Genome-wide computational identification and manual annotation of human long noncoding RNA genes. RNA.

[24] Trimarchi T, Bilal E, Ntziachristos P, Fabbri G, Dalla-Favera R, Tsirigos A, Aifantis I (2014). Genome-wide mapping and characterization of Notch-regulated long noncoding RNAs in acute leukemia. Cell.

[25] McLean MH, El-Omar EM (2014). Genetics of gastric cancer. Nature reviews Gastroenterology & hepatology.

[26] Sahin IH, Hassan MM, Garrett CR (2014). Impact of non-steroidal anti-inflammatory drugs on gastrointestinal cancers: current state-of-the science. Cancer letters.

[27] Wang F, Meng W, Wang B, Qiao L (2014). Helicobacter pylori-induced gastric inflammation and gastric cancer. Cancer letters.

[28] Kim J, Cho YA, Choi WJ, Jeong SH (2014). Gene-diet interactions in gastric cancer risk: A systematic review. World journal of gastroenterology: WJG.

[29] Tseng CH, Tseng FH (2014). Diabetes and gastric cancer: the potential links. World journal of gastroenterology: WJG.

[30] D'Elios MM, Montecucco C, de Bernard M (2007). VacA and HP-NAP, Ying and Yang of Helicobacter pylori-associated gastric inflammation. Clinica chimica acta.

[31] Monteleone G, Del Vecchio Blanco G, Palmieri G, Vavassori P, Monteleone I, Colantoni A, Battista S, Spagnoli LG, Romano M, Borrelli M, MacDonald TT, Pallone F (2004). Induction and regulation of Smad7 in the gastric mucosa of patients with Helicobacter pylori infection. Gastroenterology.

[32] Hosseini ME, Oghalaie A, Habibi G, Nahvijoo A, Hosseini ZM, Tashakoripoor M, Mohammadi M (2010). Molecular detection of host cytokine expression in Helicobacter pylori infected patients via semi-quantitative RT-PCR. Indian journal of medical microbiology.

[33] Cover TL, Peek RM (2013). Diet, microbial virulence, and Helicobacter pylori-induced gastric cancer. Gut microbes.

[34] Loh JT, Friedman DB, Piazuelo MB, Bravo LE, Wilson KT, Peek RM, Correa P, Cover TL (2012). Analysis of Helicobacter pylori cagA promoter elements required for salt-induced upregulation of CagA expression. Infection and immunity.

