# Supplementary material for: Lnc-SGK1 induced by Helicobacter pylori infection and highsalt diet promote Th2 and Th17 differentiation in human gastric cancer by SGK1/Jun B signaling
Source: Oncotarget. 2016 Mar 1;7(15):20549–60. doi: 10.18632/oncotarget.7823 (PMC4991474; doi:10.18632/oncotarget.7823)
Supplement: Supplementary file 1 [file oncotarget-07-20549-s001.pdf]

# SUPPLEMENTARY FIGURES

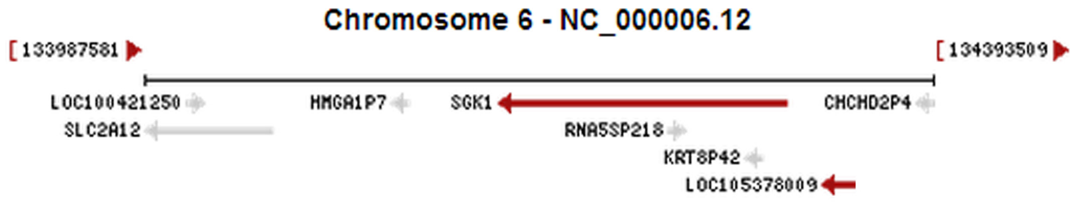


**Supplementary Figure S1: Chromosomal location of SGK1 and lnc-SGK1.**

0 Jun B was predicted in:

| | | | | | |
|---|---|---|---|---|---|
| Sequence | TGACTCA | TGACTTA | TGACTTA | TGTGTCA | TAAGTCA |
| | 325 331 | 563 569 | 625 631 | 1496 1502 | 1698 1704 |
| Dissimilarity | 0.00% | 1.83% | 1.83% | 2.14% | 1.83% |
| RE equally | 0.12207 | 0.36621 | 0.36621 | 0.36621 | 0.36621 |
| RE query | 0.12844 | 0.39838 | 0.39838 | 0.39838 | 0.39838 |

**Supplementary Figure S2: Detailed binding site of JUN B in the promoter region of lnc-SGK1.**
